# Characterization of Cellulase-Treated Fibers and Resulting Cellulose Nanocrystals Generated through Acid Hydrolysis

**DOI:** 10.3390/ma11081272

**Published:** 2018-07-24

**Authors:** Dawit Beyene, Michael Chae, Jing Dai, Christophe Danumah, Frank Tosto, Abayneh Getachew Demesa, David C. Bressler

**Affiliations:** 1 Department of Agricultural, Food and Nutritional Science, University of Alberta, Edmonton, AB T6G 2P5, Canada; dbeyene@ualberta.ca (D.B.); mchae@ualberta.ca (M.C.); janetdai100@outlook.com (J.D.); 2 Biomass Conversion and Processing Technologies, InnoTech Alberta, Edmonton, AB T6N 1E4, Canada; Christophe.Danumah@innotechalberta.ca (C.D.); Frank.Tosto@innotechalberta.ca (F.T.); 3 School of Engineering Science, Lappeenranta University of Technology, P.O. Box 20, FI-53851 Lappeenranta, Finland; abitij@gmail.com

**Keywords:** acid hydrolysis, cellulase, cellulose nanocrystals, cellulose hydrolysis, crystallinity, enzymatic treatment, particle size, zeta potential

## Abstract

Integrating enzymatic treatment and acid hydrolysis potentially improves the economics of cellulose nanocrystal (CNC) production and demonstrates a sustainable cellulosic ethanol co-generation strategy. In this study, the effect of enzymatic treatment on filter paper and wood pulp fibers, and CNCs generated via subsequent acid hydrolysis were assessed. Characterization was performed using a pulp quality monitoring system, scanning and transmission electron microscopies, dynamic light scattering, X-ray diffraction, and thermogravimetric analysis. Enzymatic treatment partially reduced fiber length, but caused swelling, indicating simultaneous fragmentation and layer erosion. Preferential hydrolysis of less ordered cellulose by cellulases slightly improved the crystallinity index of filter paper fiber from 86% to 88%, though no change was observed for wood pulp fibre. All CNC colloids were stable with zeta potential values below −39 mV and hydrodynamic diameters ranging from 205 to 294 nm. Furthermore, the temperature for the peak rate of CNC thermal degradation was generally not affected by enzymatic treatment. These findings demonstrate that CNCs of comparable quality can be produced from an enzymatically-mediated acid hydrolysis biorefining strategy that co-generates fermentable sugars for biofuel production.

## 1. Introduction

Cellulose nanocrystals (CNCs) are nanomaterials with potential application as reinforcement fillers, additives, or templates for the development of renewable and high-performance composite materials [1]. CNCs can be isolated from various cellulosic feedstocks, with variable dimensions depending on the source material (Table 1). In general, the nanoscale size and high crystallinity of CNCs allow the particles to exhibit a high surface area and excellent mechanical properties, respectively [2,3]. In addition, CNCs have low density, ideal for the reinforcement of light weight composite materials that can substitute non-renewable parts used in the automotive and aviation industries [4]. As additives, CNCs can improve the rheological properties of products in the oil drilling, aviation, pharmaceutical, food, cosmetics, and paint industries [5,6,7].

Acid hydrolysis with sulfuric (H_2_SO_4_) [14,15,16] or hydrochloric (HCl) acid [17,18] is the most common method for isolating CNCs [19]. The strong acid degrades the readily accessible glycosidic bonds in the less ordered cellulose chains, whereas the tightly packed highly crystalline region remains recalcitrant as it limits the penetration of acid and water [20]. Therefore, acid hydrolysis stabilizes over time and releases homogenous size fragments, which are thought to correspond to the native size of the crystals with the highest level of ordering [21]. The type of acid used for extracting CNCs can affect the characteristics of the final CNC product. When sulfuric acid is used, sulphate groups from the acid esterifies the free hydroxyl groups on the surface of the CNC [22]. Negative charges on the surface create an electrostatic repulsion force that maintains a stably dispersed CNC colloid in non-acidic aqueous solutions [23]. In contrast, HCl-hydrolyzed CNCs have relatively low concentrations of strong and weak acid groups bound on the surface that allow the crystals to aggregate and flocculate due to van der Waals attraction in aqueous solutions [17]. This is relevant for reinforcement applications because when particles self-aggregate, the high surface area that allow CNCs to bond with the matrix and impart strong mechanical properties is compromised [3].

High production cost is one of the issues limiting the incorporation of CNC into commercial products [1]. Efforts have been made to improve yields from acid hydrolysis and reduce energy requirements of mechanical processes by introducing a cellulase enzymatic treatment process [24,25,26,27]. Three classes of cellulases work synergistically to hydrolyze cellulose by first opening up the chain with endoglucanases, followed by the more aggressive exoglucanase hydrolysis of the polymer to cellobiose sugar, which is solubilized to glucose with *β*-glucosidase [28]. Modifications in the structure by enzymatic hydrolysis of the less ordered cellulose to glucose facilitates the CNC isolation processes. However, few studies have integrated cellulose nanoparticle production with co-generation of fermentable sugars, whereby significant yields from enzymatic hydrolysis can generate additional revenue. Zhu et al. [29] and Song et al. [30] treated wood pulp with cellulases to recover sugars for ethanol fermentation and recalcitrant crystalline solid for isolating cellulose nanofibrils (0.5–1 µm long and 5–20 nm in diameter) via mechanical homogenization and sonication, respectively. We reported our findings on the co-generation of CNCs and sugars from an enzymatically-mediated acid hydrolysis process [31]. The study demonstrated the potential for recovering fermentable sugars with variable yields (24–56 wt %) by manipulating the enzymatic hydrolysis period. CNC recovery from acid hydrolysis of enzyme-treated feedstock nearly doubled compared with the untreated feedstock. The enzymatic treatment was suggested to concentrate the crystalline cellulose fractions as less ordered cellulose was preferentially degraded in the feedstock. Further characterization studies are required to verify this interpretation. These findings from the enzymatically-mediated CNC production process demonstrate that the cost of acid hydrolysis can be reduced and a sustainable cellulosic ethanol production strategy can be developed. In order to further improve the system and fully realize these prospects, understanding the effect of the enzymatic treatment on the fiber and assessing the quality of CNCs isolated from the CNC/fermentable sugar co-production as a biorefining strategy are vital.

In the present study, enzyme-treated celluloses and CNCs generated from respective feedstocks via acid hydrolysis were characterized with the objectives of (1) deciphering the mechanism of enzymatic degradation from scanning electron microscopy, pulp quality monitoring system and X-ray diffraction pattern analyses, and (2) assessing the effect of enzymatic treatment on the hydrodynamic diameter, dimensions (length, and width), degree of crystallinity, and colloidal stability of CNCs from dynamic light scattering, transmission electron microscopy, X-ray diffraction pattern, and zeta potential analyses, respectively.

## 2. Materials and Methods

Whatman No.1 filter paper (Fisher Scientific Company, Whitby, ON, Canada) and northern bleached hardwood kraft pulp (provided by Alberta Pacific Forest Industries Inc., Al-Pac, Edmonton, AB, Canada) were studied as model and industrial (CNC pilot plant at InnoTech Alberta, Edmonton, AB, Canada) cellulosic feedstocks, respectively. Samples of washed and freeze-dried fibers that were treated with a cellulase cocktail (NS 51129, Novozymes A/S, Bagsvaerd, Denmark) and CNCs isolated by acid hydrolysis (64% H_2_SO_4_ *w*/*w*, 45 °C, 2 h) generated from experiments described in our previous study [31] were characterized. Sodium chloride was purchased from Fisher Scientific Company (Whitby, ON, Canada).

Retsch ZM 200 Ultra Centrifugal Mill (Newton, PA, USA) was used to generate powder samples for the X-ray powder diffraction (XRD) study. Rigaku Ultima IV diffractometer (Rigaku Corporation, Tokyo, Japan), at the nanoFAB fabrication and characterization center (University of Alberta), was used to analyze the degree of crystallinity from XRD spectra. Pulp quality monitoring system (PQM 1000, Metso Sweden AB, Sundsvall, Sweden) was used to measure fiber dimension and length distribution. Messmer pulp disintegrator MK111C (Messmer Instruments Ltd., Gravesend, UK) was used to disintegrate the original, untreated feedstock. Malvern Zetasizer Nano-ZS (Malvern Panalytical Ltd., Almelo, Netherlands) was used for hydrodynamic diameter and surface charge analyses. Access to the disintegrator, PQM system and Zetasizer were kindly facilitated by InnoTech Alberta (Edmonton, AB, Canada). Leica EM SCD005 (Leica Microsystems Inc., Wetzlar, Germany) was used to coat samples with carbon. Scanning electron microscope (SEM) (Carl Zeiss AG, Oberkochen, Germany) images were generated on a Zeiss Sigma 300 Variable Pressure-Field Emission (VP-FESEM) (Department of Earth and Atmospheric Sciences, University of Alberta). Philips/FEI, Morgagni 268 transmission electron microscope (TEM, Hillsboro, OR, USA), at the Advanced Microscopy Facility (Department of Biological Sciences, University of Alberta) was used for image analysis of CNCs. Thermogravimetric Analyzer Q50 (Thermal Analyzers, Newcastle, DR, USA), at the Lipid Chemistry Group Laboratory (Department of Agricultural, Food and Nutritional Science, University of Alberta), was used to for evaluating thermal stability.

### 2.1. Fiber Structure and Dimensions

#### 2.1.1. SEM Imaging

Enzyme-treated fiber samples were soaked in water (25 mg in 2.5 mL H_2_O, 1% *w*/*v*) and vortexed at high speed. A drop of the fiber suspension was mounted onto stubs and dried in a desiccator overnight. Samples were carbon coated and analyzed on SEM, whereby electrons were accelerated at 10 kV and images were magnified 1000×.

#### 2.1.2. Image Analysis on Pulp Quality Monitoring System

Original, untreated filter paper and wood pulp samples (0 h enzymatic treatment) were pre-soaked in water (24 g in 2 L H_2_O, 1.2% *w*/*v*) for 4 h. Since the fibers are intertwined in these feedstocks, a pulp disintegrator operating at 3000 rpm for 1 min was used to separate individual fibers. Enzyme-treated fibers were in powder form; hence, separation was simply conducted by stirring a fiber suspension (2 g in 200 mL H_2_O, 1% *w*/*v*) at low speed with a magnetic stirrer overnight. 

The fiber suspensions were further topped up with water to a final volume of 600 mL and loaded into the PQM system to analyze the average length, width, and length distribution. The system measured the dimensions of fibers (70,000 to 240,000 counts) passing through a glass cell, based on image analysis of transmitted variable light beam distribution [32].

### 2.2. CNC Particle Size and Zeta Potential Measurements

#### 2.2.1. Dynamic Light Scattering

The hydrodynamic diameter and the colloidal stability of CNCs were estimated from dynamic light scattering and zeta potential analyses on a Malvern Zetasizer Nano-ZS.

##### Hydrodynamic Diameter

Small particles in colloid exhibit Brownian movement. When a colloid sample is scanned with a laser, the motion causes light scattering, whereby the intensity fluctuates over time. A translation diffusion coefficient (*D_t_*) can be derived from a correlation function (mathematical functions not shown here). This coefficient can be used to calculate the hydrodynamic diameter (*d_H_*) of spherical particles using Stoke-Einstein relation in Equation (1):

(1) $$d_{H}=\frac{kT}{3\pi \eta D_{t}}\;\;$$

where *k* is the Boltzmann’s constant, *T* is the absolute temperature, and η is the viscosity of the colloid [33,34]. 

CNC colloid was diluted with sodium chloride (10 mM NaCl) solution to provide final CNC and salt concentrations of 0.05 wt % and 5 mM NaCl, respectively. Salt was added to prevent overestimation of hydrodynamic diameter by increasing the ionic strength to reduce the thickness of the electric double layer surrounding the CNC particles [35].

Colloid samples were loaded on a cuvette and scanned with a 633 nm light beam generated from a 4.0 mW He-Ne laser. The intensity of scattered light was measured with an avalanche photodiode detector positioned at 173° to estimate the light scattering intensity fluctuations.

##### Colloid Stability

Zeta potential is a good indicator of the stability of colloids based on electrostatic repulsive forces on the surface charges. When a colloid is subjected to an electric field, the particles move toward the opposite charge. While in motion, a hypothetical plane called the slipping plane is formed within the electric double layer between the moving charged particle and the dispersant layer. The potential difference formed at this plane is called the zeta potential. In estimating zeta potential, the particle velocity is derived from the frequency shift of the scattering light. Electrophoretic mobility (µ_e_) is determined from the particle velocity (mathematical function not shown here) and is used to calculate zeta potential (ζ) from Henry’s equation:

(2) $$\zeta =\frac{{\text{µ}}_{e}3\eta }{2\varepsilon_{r}\varepsilon_{0}f\left(Ka\right)}\;\;$$

where ε_r_ is the dielectric constant of the medium, *ε*_0_ is the permittivity of vacuum, *f*(*Ka*) is Henry’s function, and η is the viscosity of the colloid [34,36]. 

An electric field was induced by an electrode (dip cell) inserted into the cuvette containing the same CNC colloid aliquot for hydrodynamic diameter analysis to estimate the frequency shift of the scattering light.

Light scattering data were automatically analyzed and computed on the built-in Zetasizer software to generate hydrodynamic diameter and zeta potential estimations.

#### 2.2.2. Particle Size from TEM Analysis

A CNC sample (freeze-dried for long term storage from an aliquot CNC colloid generated in the previous study [31]) was re-suspended in water (0.1 g in 100 mL H_2_O, 0.1% *w*/*v*) and sonicated for 15 min. A droplet of the CNC colloid was mounted on a copper grid (300 mesh) coated with formvar film (Ted Pella Inc., Redding, CA, USA) for 20–30 min. Excess liquid was removed with filter paper and the grid was negatively stained with phospho-tungstic acid (2% *w*/*v*) for 15 s. Excess stain was removed with filter paper and the copper grid was loaded on a TEM operating at 80 kV electron speed and 110,000× magnification. Length and width of well separated CNC rods (total count of 75 from all triplicates), with distinguishable tip ends and widths, were analysed using image J software (National Institute of Health, Rockville, MD, USA). A straight line tool, standardized by the scale bar generated from the TEM micrograph, was used for manual measurement of individual particles on the software. The aspect ratio of an individual CNC particle was calculated from the ratio of the longest side to the shortest side.

### 2.3. Degree of Crystallinity of Fibers and CNCs

**Figure 1:**
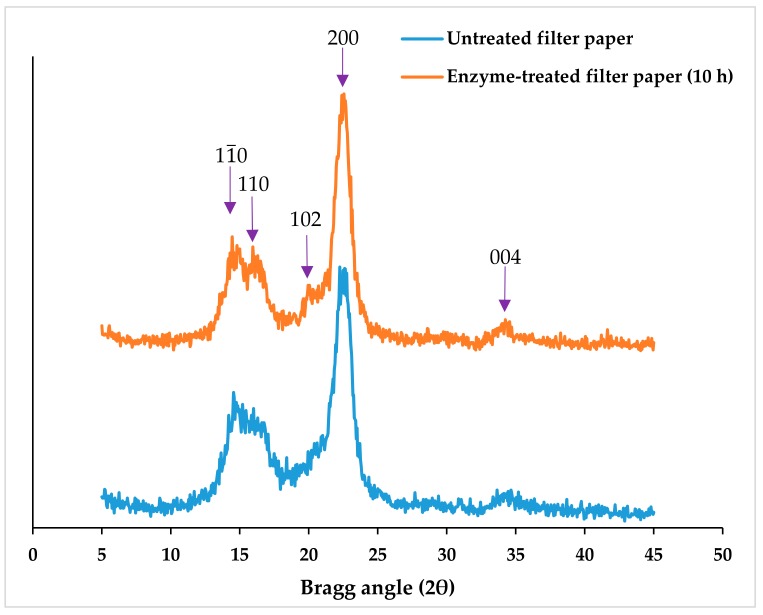
X-ray diffraction (XRD) spectra of untreated (0 h) and enzyme-treated (10 h) filter paper.

Fiber samples were powdered by milling at 8000 rpm and passed through 0.5 mm screen, whereas freeze-dried CNC was already in powdered form. The sample was scanned with an X-ray beam generated from a Cu tube (at 40 kV and 44 mA and controlled via 10 mm divergent slit), over 5°–45° Bragg angles (2θ) and at a scan speed of 2° per minute. The spectra were processed through JADE software (Jade Software Corporation Limited, Christchurch, Australia) to format and access the intensity measurements. The degree of crystallinity was assessed from the crystallinity index (%) that was calculated based on the peak height method using Equation (3) in which *I*_*am*_ is the intensity count at the tip end of the minimum valley between the 110 and 200 planes (around 2θ = 18°) (Figure 1), representing the less ordered cellulose, and *I*_*total*_ is the intensity count at the maximum height of the peak at 200 plane (2θ in the range between 22°–24°), representing both crystalline and less ordered cellulose [37,38].

(3) $$\mathrm{Crystallinity}\;\mathrm{Index}=\frac{I_{total}-I_{am}}{I_{total}}\times 100\%$$

### 2.4. CNC Thermogravimetric Analysis 

The thermal stability of CNC particles was analysed on a Thermal Analyzer Q50. Freeze-dried CNC samples from filter paper (5–17 mg) and wood pulp (3–10 mg) were heated in a furnace under nitrogen gas (60 mL/minute flow rate), with temperature increasing at a rate of 10 °C/minute from room temperature up to 500 °C. The onset of degradation temperature (°C) was identified from the intersection of an initial line tangent to the constant weight line plot and a final line tangent to the infection point, implicating weigh loss from thermal degradation. The temperature (°C) at which the maximum rate of change in weight per temperature (%/°C) was attained from the derivative plot.

### 2.5. Data Analysis

All reported data (mean ± standard deviation) represent analyses from triplicate samples, unless specified. One-way ANOVA combined with Tukey’s test, at a 95% confidence interval (CI) was calculated to compare the means on Minitab 17 and 18 software, versions 17.3.1 and 18.1, respectively (Minitab Inc., State College, PA, USA). Outliers were detected from the data set using interquartile range test.

## 3. Results and Discussion

### 3.1. Characteristics of Enzyme-Treated Fibers

#### 3.1.1. Fiber Structure and Dimension

**Figure 2:**
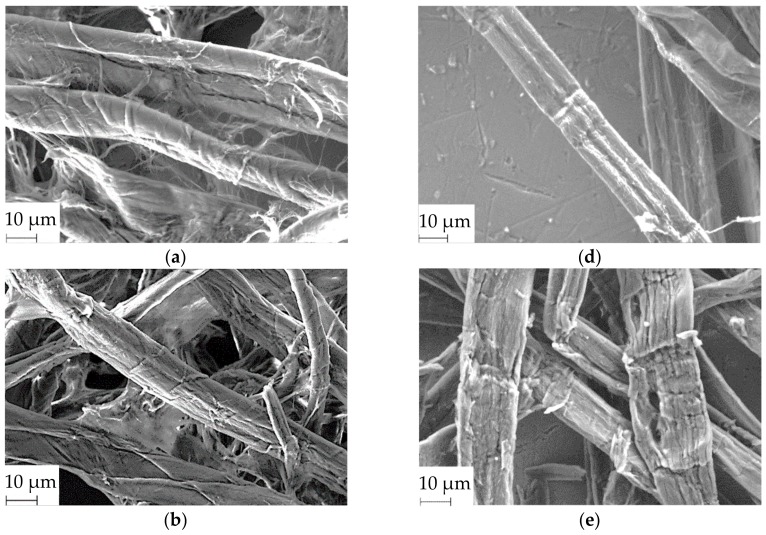

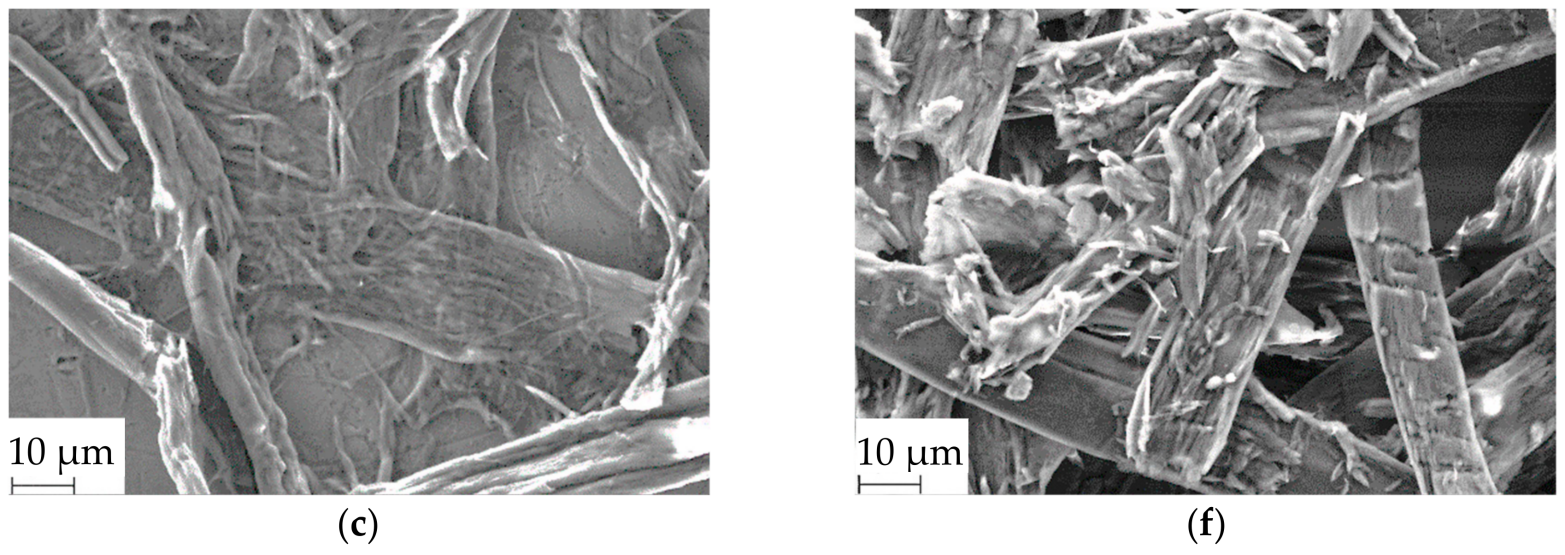
Scanning electron microscopy (SEM) micrograph of filter paper fiber, treated with cellulase for (**a**) 0 h, (**b**) 2 h, and (**c**) 10 h and wood pulp fiber treated with cellulase for (**d**) 0 h, (**e**) 2 h, and (**f**) 10 h.

SEM and pulp quality monitoring (PQM) system analyses were completed to study the structure and size of enzyme-treated fibers, respectively. Despite substantial degradation (45.4 ± 1.8% undigested solid recovered from 10 h treatment, wt % wood pulp substrate) reported in our previous study [31], fiber lengths that were up to a few millimeters in length were apparent in SEM micrographs (Figure 2c,f). This implies that the cellulases did not entirely cut the fiber length into very short fragments or fines. PQM system analysis showed that enzymatic treatment significantly reduced the average fiber length from 1.21 ± 0.10 up to 0.68 ± 0.03 mm in filter paper and 1.10 ± 0.01 up to 0.75 ± 0.06 mm in wood pulp (Table 2). No significant change was observed in the length as a function of enzyme treatment period (except at 10 h for filter paper) in both feedstocks.

**Figure 3:**
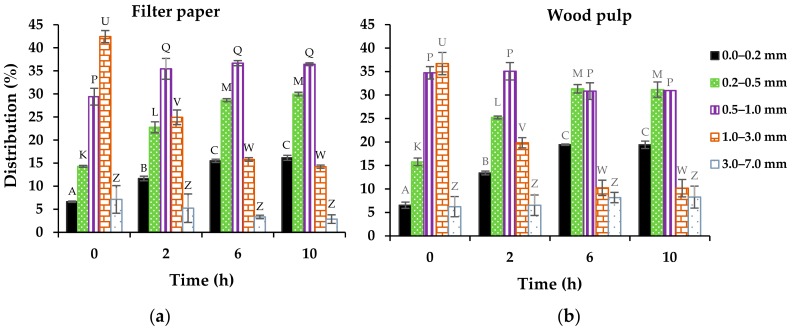
Length distribution of enzyme-treated (**a**) filter paper and (**b**) wood pulp fibers. Means with non-identical letters (in superscripts) are significantly different (*p* < 0.05) from comparisons within a given fiber length size as a function of time for each chart.

The length distribution data shows that the abundance of short fragments (0–0.2 and 0.2–0.5 mm) significantly increased as a function of enzyme hydrolysis period until six hours (Figure 3). However, these fragments only accounted for 19% of the total distribution. Fiber lengths in the range of 0.5 to 1.0 mm dominated the length distribution (29%–37%) of enzyme-treated fibers. These data agreed with the observations from SEM analysis. Conversely, the abundance of 1 mm–3 mm fibers, which was most prominent in the undigested feedstock (37%–42%), was significantly reduced (10%–16%) by enzymatic treatment until six hours. The distribution of 3.0–7.0 mm long fibers did not change throughout the enzymatic treatment period. The degradation of long fibers seven or less mm long to fragments more than three mm long was likely masked during the distribution due to the broadness of the range.

Remarkably, fiber width was not reduced even after longer treatments, with broad fibers (up to 24 µm) still visible in the SEM micrographs (Figure 2). The PQM system analysis revealed that the width of filter paper and wood pulp fibers significantly increased from 29 ± 1 to 36 ± 1 µm and 32 ± 0 to 42 ± 1 µm due to enzymatic treatment in both feedstocks (Table 2). In the case of wood pulp, a significant increase in width as a function of enzyme treatment period (until six hours) was even observed. Song et al. reported significant reduction in lengths but no changes in the diameter (several micrometers in dimension) of enzyme-treated fibers [30]. During cellulase hydrolysis, the fibers have likely undergone swelling by the action of carbohydrate-binding modules and non-hydrolytic enzymes, which induce amorphogenesis [39].

The detailed structure of individual enzyme-treated pulp fibers under SEM revealed weak junctions along the length (Figure 2e), which appeared deeper with longer enzyme treatment periods (Figure 2f). At a longer treatment time (10 h), the erosion of smaller fragments from these degradation sites were also visible, whereas some long fibers were still observed (Figure 2c,f). In summary, interesting findings were obtained from the analysis of enzyme-treated fibers, such as the prevalence of very long fibers, low abundance of very short fragments, implications of fiber swelling, and indications of progressive weakening of the fiber structure along the length. These observations suggest that during enzymatic treatment of cellulose fibers, fragmentation and peeling were simultaneously in effect, facilitated by amorphogenesis [40].

#### 3.1.2. Degree of Crystallinity of Enzyme-treated Fibers

The crystallinity indices of fibers from enzyme-treated feedstock were assessed from XRD spectra analyses based on the peak height method. The fiber crystallinity indices of enzyme-treated filter paper at 6 h (88.6% ± 0.6%) and 10 h (88.4% ± 0.4%) improved significantly, relative to the original, untreated substrate (85.9% ± 0.7%) (Table 3). The less ordered cellulose was likely preferentially hydrolyzed by the enzymes, which led to an increase in the concentration of crystalline domains. The tightly packed chains in the crystalline regions limit access and penetration not only to enzymes, but also water and even acids [20,30]. Studies have shown that the rate of enzyme hydrolysis is typically faster for less ordered cellulose relative to crystalline cellulose [41,42,43]. The improvement in fiber crystallinity indices supports the hypothesis in our previous report, which stated that CNC recovery improved because enzymatic treatment concentrates the CNC precursors as less ordered cellulose is preferentially hydrolyzed [31].

As a function of enzyme hydrolysis time, no significant difference was observed in the fiber crystallinity index of enzyme-treated filter paper feedstock (Table 3). We previously reported that there was no significant improvement in CNC yield (wt % acid hydrolyzed feedstock) from these feedstock as a function of time (except at 10 h) [31]. This finding indicates simultaneous hydrolysis of crystalline cellulose throughout the treatment period. Ramos et al. observed no changes in the fiber crystallinity index of enzyme-treated cellulose and suggested that the enzymes were peeling off the crystalline domains on the exposed surfaces [44]. The interspersed arrangement of less ordered and crystalline domains implies that amorphous cellulose embedded in the core is inaccessible [40,42]. Simultaneous amorphogenesis and gradual erosion of layers, evident from the fiber structures on SEM (Figure 2), are likely to expose the crystalline celluloses to enzymatic hydrolysis [40,45]. Therefore, enzymatic treatment, in the presence of exoglucanases that also act on crystalline cellulose [28], was not entirely selective to less ordered cellulose. In addition, cellulose with a higher degree of crystallinity was highly abundant in filter paper compared with wood pulp. This was evident from the relatively higher fiber crystallinity, the requirement of higher effective enzyme loading, and the recovery of up to 70% CNC from acid hydrolysis of enzyme-treated filter paper [31]. Despite being recalcitrant, these abundant crystalline regions are likely to be the major available substrate for enzymatic adsorption and hydrolysis. Hence, the simultaneous degradation of less ordered and crystalline cellulose has led to a constant crystallinity index as a function of enzyme treatment period.

Interestingly, enzymatic treatment did not significantly change the crystallinity index of wood pulp fiber (Table 3). This was surprising, considering the significantly large improvements up to 86% in CNC recovery (wt % acid-hydrolyzed feedstock) from the enzyme-treated wood pulp that was reported previously [31]. Conflicting findings have been reported in the literature regarding the correlation between enzyme hydrolysis and degree of crystallinity. Some studies reported that fiber cellulose crystallinity index improved due to enzymatic treatment [24,29,46]. In contrast, others studies did not find any change in fiber crystallinity indices [44,47], even after 90% hydrolysis, using the more sensitive (CP/MAS) ^13^C-nuclear magnetic resonance (NMR) spectroscopy analysis [43]. After investigating multiple XRD and NMR analyses, Park et al. concluded that in order to pose inferences about preferential hydrolysis, the less ordered cellulose in the substrate must be severely degraded [45]. In the present study, the most aggressive treatment (45.4 ± 1.8 wt % undigested solid recovered from 10 h hydrolysis [31]) may not have reached the level of severity required to interpret fiber crystallinity. In addition, the limitations in the XRD data interpretations via the empirical method could mask the changes in fiber degree of crystallinity. In Equation (3), the 200 plane is the only position that was accounted for in the crystalline cellulose domains. However, four other planes (1$\bar{1}$0, 110, 102, and 004) also displayed characteristic XRD peaks in the spectra (Figure 1) [45]. The improvement in the crystalline fractions of cellulose composition in these regions may have been overlooked. 

### 3.2. Characteristics of CNCs Isolated from Enzyme-treated Fibers via Acid Hydrolysis

#### 3.2.1. Particle Size 

##### Dynamic Light Scattering Analysis

**Figure 4:**
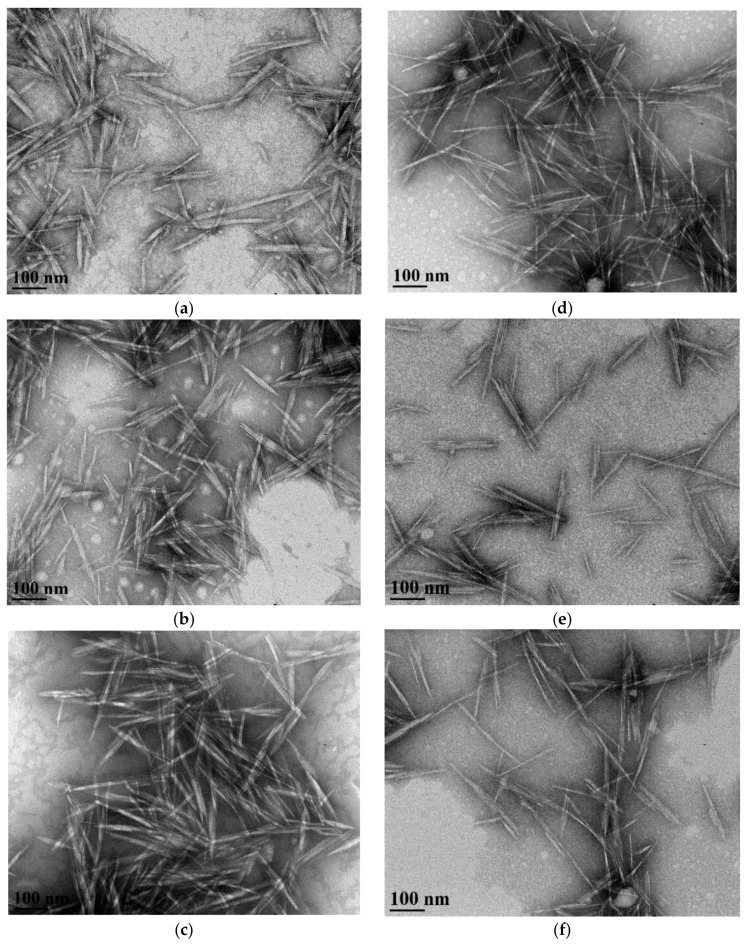
Transmission electron microscopy (TEM) micrographs of CNC isolated from: filter paper enzyme-treated for (**a**) 0 h, (**b**) 2 h, and (**c**) 10 h and wood pulp enzyme-treated for (**d**) 0 h, (**e**) 2 h, and (**f**) 10 h.

The size of the nano-particles was estimated from light scattering analysis based on the Stoke-Einstein relation Equation (1). However, this equation works best for measuring the diameter of a spherical particle in a colloid as a function of the translational diffusion coefficient. CNCs have a rod/needle shape structure with two unequal dimensions of length and width (TEM image of CNC, Figure 4). Boluk and Christophe compared the length estimations from the Stoke-Einstein equation with TEM image measurements [35]. A substantial difference was found in the length, most likely due to the influence of the width on the overall diffusion rate. The translation diffusion rate parallel to the longitudinal axis is known to be faster than the motion perpendicular to it. Hence, the estimation from dynamic light scattering analysis is an interdependent parameter referred to as hydrodynamic diameter. Yet, this measurement is still valuable for relative comparison of CNC particle size. 

The average hydrodynamic diameter of enzyme-treated filter paper and wood pulp ranged from 221 ± 34 to 294 ± 51 nm and 196 ± 31 to 245 ± 63 nm, respectively (Table 4). The effect of enzymatic treatment was only observed on the filter paper feedstock with a significant decrease in the average hydrodynamic diameter of CNC particles starting from two hours of enzyme-treated cellulose. The CNC crystals in filter paper were possibly substantially more accessible to cellulase degradation, which reduced the dimension of the CNC precursors. This is likely due to the relatively higher degree of crystallinity (85.9% ± 0.7%) of filter paper fibers relative to wood pulp (76.6% ± 0.8%). Enzymatic hydrolysis had no significant effect as a function of enzymatic treatment period. Beltramino et al. studied the characteristics of CNCs from cellulase-treated cotton linter and reported that the particle size did not change due to enzymatic hydrolysis [24].

##### TEM Image Analysis

The lengths and widths of the CNCs were measured from analysis of TEM micrographs (Figure 4). The average lengths of CNCs isolated from untreated (0 h) filter paper and wood pulp feedstock were 116 ± 30 and 113 ± 30 nm, respectively. Enzymatic treatment did not have any significant effect on CNC lengths in both feedstocks, except at two hours for filter paper, with sizes ranging from 123 ± 45 to 138 ± 44 nm for filter paper CNC and 109 ± 33 to 126 ± 42 nm for wood pulp CNC (Table 5). As expected, these length measurements were substantially different from the hydrodynamic diameter estimations as the latter is only an indirect estimation, as discussed previously. The widths of CNCs from enzyme-treated filter paper were uniform (9.5 ± 3.0 to 10.2 ± 3.0 nm) and similar with CNCs from untreated (0 h) filter paper (8.8 ± 2.5 nm), except at two hours. However, enzymatic treatment significantly decreased the width of CNC wood pulp from 9.2 ± 3.3 nm (untreated, 0 h) to up to 6.8 ± 2.1 (six hours enzyme-treated). Studies in the literature reported the particle size of CNC from TEM analysis of wood and cotton as ranging from 70 to 300 nm in length and 4 to 20 nm in width [8,9,13,23,49,50,51,52]. Siqueira et al. isolated CNC by acid hydrolysis of enzyme-treated and mechanically sheared pulp with particle size of 230–250 nm in length and 7 nm wide [53].

The aspect ratios calculated from the ratio of length to width of filter paper CNCs ranged from 12.0 ± 3.7 to 15.1 ± 7.0 nm, with no significant change in size due to enzymatic treatment (Table 5). No clear trend was observed in the aspect ratios of wood pulp CNC, which ranged from 13.4 ± 5.9 to 19.0 ± 6.1 nm. The aspect ratios of CNC from wood pulp were relatively lower than reports from Beck-Candanedo et al. (Table 1) [8]. Bias in the selection of particles from the TEM micrograph may have affected the average length and width data. Despite the repulsive forces from the negative surface charge, self-aggregation was apparent on the copper grid (Figure 4), as previously reported in other studies [22,51]. In addition, CNC particles isolated from cotton and wood are likely to form more aggregates compared with crystals from other sources such as tunicate [13,50]. In the present study, we observed that longer particles aggregated more than smaller size particles. This is most likely due to the relatively higher surface area for hydrogen bond formation. Therefore, the preferential selection of shorter length particles due to the characteristic dispersion probably led to an overall underestimation of the length and hence lowered the aspect ratio values. Aspect ratios were also calculated from the dataset without removing outliers to retain the longer CNC particle sizes, assuming that these particles were not apparent in the micrograph due to experimental error or by chance. However, no substantial change in the value of the aspect ratio and interpretation were observed.

#### 3.2.2. Zeta Potential Analysis

The stability of a colloid due to electrostatic repulsive forces of the charged surfaces was assessed from zeta potential measurement. The zeta potential values of CNC colloids extracted from filter paper (−40.5 ± 2.5 to −43.8 ± 0.8 mV) and wood pulp (−38.9 ± 2.7 to −42.6 ± 3.5 mV) (Table 4) were above the minimum threshold (±30 mV) for electrostatic stability considering their absolute values [54]. Studies have reported zeta potential values of −15 mV from controlled microbial hydrolysis of filter paper [55], −25 to −31 mV from acid hydrolysis of cotton fibers [9], −31 mV from enzymatic treatment of recycled paper [56], and −52 to −58 mV from acid hydrolysis of filter paper [35]. Large zeta potential values do not always guarantee stability, as strong van der Waals attractive forces may cause agglomeration [34]. Based on visual observation of some samples, the colloids in the present study did not show precipitation for several days and hence support the stability implications from the estimated zeta potential values.

Similar to the particle size estimations, only the zeta potential values of filter paper feedstock were affected by enzymatic treatment (Table 4). The zeta potential values significantly increased from −40.5 ± 2.5 (0 h) to −43.8 mV (with standard deviation of ± 0.8 for 6 h and ± 0.6 for 10 h). The enzymes likely altered the hydroxyl groups distribution on the CNC surface, which could affect the esterification of the sulfate group and hence the charge density on the surface [24].

#### 3.2.3. CNC Degree of Crystallinity 

Acid hydrolysis improved the crystallinity indices of cellulose isolated from filter paper and wood pulp celluloses (0 h, untreated) from 85.9 ± 0.7% to 88.2 ± 0.5% and 76.6 ± 0.8% to 80.2 ± 0.9%, respectively (Table 3). This implies that the strong acid substantially hydrolyzed the less ordered celluloses during CNC isolation. The crystallinity index of filter paper CNC, in the range of 86.0 ± 0.6 to 88.9% ± 0.3%, was much higher compared with wood pulp CNC (in the range of 78.5% ± 2.2% to 80.9% ± 0.8%). This implies that the level of ordering of the crystalline fractions in the filter paper CNC is higher relative to wood pulp, suggesting that acid hydrolysis of less ordered cellulose does not guarantee a uniform CNC crystallinity index from any given feedstock. CNC degree of crystallinity from a particular feedstock is a function of the level of crystalline cellulose chain ordering in the native structure. The substantially higher fiber crystallinity index of the original, untreated filter paper cellulose (85.9% ± 0.7%) relative to wood pulp (76.6% ± 0.8%) also supports this suggestion. Variable CNC crystallinity indices, from XRD analysis of acid hydrolyzed cotton (87%–91%), rice straw (86%–91%), wood (75%–85%), bagasse (73%) ,and coconut fiber (66%) have been reported in the literature [9,10,12,22,52,57].

The crystallinity index of enzyme-treated fibers at a given enzymatic treatment period (for instance, two hours) was compared with the crystallinity index of CNCs isolated from acid hydrolysis of the same fiber (two hours of enzyme-treated feedstock). In the case of the filter paper feedstock, no substantial differences in crystallinity indices of fibers and CNCs were identified in such comparisons across the same treatment period (Table 3). It is possible that a value close to the maximum achievable degree of crystallinity of fiber was reached due to the substantial enzymatic degradation of poorly ordered cellulose, even within two hours. XRD analysis may lack the sensitivity to detect subtle changes in the degree of crystallinity of CNCs due to the limitations discussed previously. During acid hydrolysis, the acid was mainly acting on the available highly ordered CNC precursor. The fiber particles were evidently reduced from millimeters and micrometer size lengths and widths, respectively (Table 2 and Figure 2) to nanoscale-sized CNC particles (Table 5 and Figure 4). In the process, the acid also likely dissolved part of this cellulose chain to oligosaccharides or monomer glucose units given the substantial loss of cellulose. We previously reported 64%–70% CNC recovery from acid hydrolysis of enzyme-treated filter paper (2–10 h) [31]. In the absence of substantial composition of less ordered cellulose, the chain ends of the CNC precursors and/or isolated CNCs are likely attacked with hydronium ions during the residence time. Hence, cellulose dissolution was probably in effect on the easily accessible chain ends. With prolonged exposure time to acid, reduction in CNC size has been previously reported in the literature [8,16].

In contrast, the CNC crystallinity indices from enzyme-treated wood pulp improved by up to five units (%), relative to the enzyme-treated wood pulp fiber across the same time period (Table 3). Even though CNC precursors are concentrated in the enzyme-treated wood pulp from preferential hydrolysis, evident from improvement in the CNC recovery wt % acid hydrolyzed wood pulp [31], the feedstock still had a substantial composition of less ordered cellulose. As discussed previously, this is likely due to the inaccessibility of less ordered cellulose chains that are trapped in the core structure [40,42].

The CNC crystallinity indices did not show any significant difference due to enzymatic treatment or as a function of time in both feedstocks, except for CNC from six hours of enzyme-treated filter paper (Table 3). This implies that the degree of crystallinity of CNC is a function of the native crystal perfection in the acid recalcitrant cellulose. We suggest that, despite some evident crystalline cellulose degradation in the fiber discussed previously, enzymatic treatment does not change the quality of the CNC precursor crystals.

#### 3.2.4. Thermal Stability

Thermogravimetric analysis was completed to assess the degradation profile of CNC particles as a function of temperature. Identification of the onset temperature of degradation was challenging as the change in slope of the weight (%) for majority of the samples was ambiguous. The derivative plots also did not show clearly distinguishable peaks, which led to highly subjective sketching of the final tangential line. Hence, the estimations of the onset temperature of degradation could not be used and reported for this comparative study. Conversely, the derivative plots distinctly provided the temperature profile for the maximum rate of weight loss (%/°C) of CNCs from the steepest peaks. Accordingly, the peak degradation temperatures for filter paper and wood pulp CNCs were between 290 ± 32 to 311 ± 1 °C and 301 ± 2 to 305 ± 1 °C, respectively (Table 6). Yildirim et al. reported a maximum rate of CNC weight loss at 247.9 °C [58]. Enzymatic treatment had no significant effect on this rate in both feedstocks (except at 10 h).

## 4. Conclusions

Enzymatic treatment of the filter paper and wood pulp decreased the fiber length without complete fragmentation, but with gradual swelling and erosion of layers. The degree of crystallinity of filter paper fiber improved due to enzymatic treatment revealing some level of preferential hydrolysis of less ordered cellulose. The limitations in the analysis of fiber crystallinity index may have masked the same effect in wood pulp. Enzymatic treatment reduced the hydrodynamic diameter and improved the stability of CNC colloids isolated from acid hydrolysis of enzyme-treated filter paper due to the modification of the abundant and accessible crystals in this feedstock. The thermal stability and degree of crystallinity of CNCs were generally not affected due to the enzymatic treatment. Enzymatic treatment of filter paper can generate a highly crystalline feedstock equivalent to what can be achieved with acid hydrolysis. Overall, this study demonstrated that enzymatic treatment did not substantially affect the quality of the CNC generated from acid hydrolysis. The enzymatically-mediated CNC production process promises to be a potentially viable biorefining strategy for the co-production of fermentable sugars and CNC without compromising the characteristics of the biomaterial.

## Figures and Tables

**Table 1:** Length and width of cellulose nanocrystals (CNCs) from different sources.

Feedstock	Length (nm)	Width (nm)	Aspect Ratio	Reference
Wood	105–147	4.5–5	23–31	[8]
Cotton	130–180	10–14	NR	[9]
Coconut fiber	177–218	5–7	35–44	[10]
Rice straw	117–270	11–30	9–11	[11]
Bagasse	250–480	20–60	NR	[12]
Bacteria	1103	14	94	[13]
Tunicate	1187	9	148	[13]

NR—not reported.

**Table 2:** Average length and width of enzyme-treated filter paper and wood pulp based on pulp quality monitoring analysis.

Enzymatic Treatment (h)	Filter Paper		Wood Pulp	
	Length (mm)	Width (μm)	Length (mm)	Width (μm)
0	1.21 ± 0.10 ^A^	29 ± 1 ^a^	1.10 ± 0.01 ^P^	32 ± 0 ^p^
2	0.90 ± 0.11 ^B^	33 ± 1 ^b^	0.88 ± 0.07 ^Q^	37 ± 1 ^q^
6	0.71 ± 0.01 ^B,C^	36 ± 1 ^b^	0.75 ± 0.07 ^Q^	42 ± 1 ^r^
10	0.68 ± 0.03 ^C^	35 ± 2 ^b^	0.75 ± 0.06 ^Q,^*	41 ± 1 ^r^

Means with non-identical letters (in superscripts) within each column are significantly different (*p* < 0.05). * Calculated from duplicates after removing an outlier.

**Table 3:** Degree of crystallinity of enzyme-treated cellulose chains and cellulose nanocrystal (CNC) isolated from filter paper and wood pulp via acid hydrolysis.

Enzymatic Treatment (hrs)	Crystallinity Index (%)			
	Enzyme-Treated Fiber		CNC Isolated via Acid Hydrolysis	
	Filter Paper	Wood Pulp	Filter Paper	Wood Pulp
0	85.9 ± 0.7 ^A^	76.6 ± 0.8 ^P^	88.2 ± 0.5 ^A^	80.2 ± 0.9 ^P^
2	87.3 ± 1.0 ^A,B^	75.9 ± 1.8 ^P^	87.6 ± 0.9 ^A,B^	80.7 ± 3.5 ^P^
6	88.6 ± 0.6 ^B^	76.0 ± 1.4 ^P^	86.0 ± 0.6 ^B^	78.5 ± 2.2 ^P^
10	88.4 ± 0.4 ^B,^*	78.0 ± 1.8 ^P^	88.9 ± 0.3 ^A,^*	80.9 ± 0.8 ^P^

Means with non-identical letters (in superscripts) within each column are significantly different (*p* < 0.05). * Calculated from duplicates.

**Table 4:** Particle size and colloidal stability of CNCs isolated from enzyme-treated filter paper and wood pulp based on dynamic light scattering and zeta potential analyses.

Enzymatic Treatment (h)	Filter Paper			Wood Pulp		
	Average Hydrodynamic Diameter (nm) ^†^	Intensity Abundance (%)	Zeta Potential (mV)	Average Hydrodynamic Diameter (nm)	Intensity Abundance (%)	Zeta Potential (mV)
0	294 ± 51 ^A^	81 ± 12	–40.5 ± 2.5 ^a^	245 ± 63 ^P^	80 ± 8	–39.8 ± 3.6 ^p^
2	229 ± 53 ^B^	87 ± 11	–41.9 ± 1.5 ^a,b^	225 ± 12 ^P^	96 ± 4	–42.6 ± 1.4 ^p^
6	213 ± 9 ^B^	94 ± 4	–43.8 ± 0.8 ^b^	243 ± 45 ^P^	83 ± 12	–42.6 ± 3.5 ^p^
10	209 ± 14 ^B,^*	99 ± 2*	–43.8 ± 0.6 ^b,^*	205 ± 16 ^P^	90 ± 5	–38.9 ± 2.7 ^p^

Means with non-identical letters (in superscripts) within each column are significantly different (*p* < 0.05). ^†^ Average diameter of the peak showing the highest abundance intensity. * Calculated from duplicates. The conductivity, pH, and temperature of the colloid were 0.7 to 0.9 mS/cm, 4.6 to 4.8, and 25 °C, respectively. The polydispersity indices ranged from 0.3 to 0.6, which measures the broadness of the size distribution. Colloid with an index value below 0.05 is monodisperse, below 0.08 is nearly monodisperse, 0.08–0.7 is midrange value, and above 0.7 is a broad distribution [48]. Data represent mean ± standard deviation of analytical triplicates (processed by the instrument) of treatment triplicates.

**Table 5:** Particle size of CNC isolated from enzyme-treated filter paper and wood pulp based on transmission electron microscopy (TEM) micrograph analysis.

Enzymatic Treatment (h)	Filter Paper			Wood Pulp		
	Length (nm)	Width (nm)	Aspect Ratio *	Length (nm)	Width (nm)	Aspect Ratio
0	116 ± 30 ^A^	8.8 ± 2.5 ^a^	13.2 ± 3.6 ^E,F^	113 ± 30 ^P,Q^	9.2 ± 3.3 ^p^	13.4 ± 5.1 ^W^
2	138 ± 44 ^B^	10.2 ± 3.0 ^b^	13.5 ± 3.8 ^E,F^	126 ± 42 ^P^	6.8 ± 2.1 ^r^	18.8 ± 5.4 ^X^
6	123 ± 45 ^A,B^	9.8 ± 3.0 ^a,b^	12.0 ± 3.7 ^E^	109 ± 33 ^Q^	7.9 ± 1.9 ^q^	14.0 ± 4.4 ^W^
10	134 ± 40 ^A,B,^*	9.5 ± 3.1 ^a,b,^*	15.1 ± 7.0 ^F,^*	123 ± 32 ^P^	7.6 ± 2.3 ^q,r^	17.5 ± 7.3 ^X^

Means with non-identical letters (in superscripts) within each column are significantly different (*p* < 0.05). * Calculated from duplicates.

**Table 6:** Rate of CNC weight loss as a function of temperature.

Enzymatic Treatment (h)	Temperature for Maximum Rate of Weight Loss %/°C	
	Filter Paper (°C)	Wood Pulp (°C)
0	290 ± 32 ^A^	301 ± 2 ^P^
2	310 ± 1 ^A^	303 ± 0 ^P,Q^
6	311 ± 1 ^A^	302 ± 1 ^P,Q^
10	310 ± 2 ^A^	305 ± 1 ^Q^

Means with non-identical letters (in superscripts) within each column are significantly different (*p* < 0.05).
